# Expanding the Obesity Research Paradigm to Reach African American Communities

**Published:** 2007-09-15

**Authors:** Shiriki Kumanyika, Melicia C. Whitt-Glover, Tiffany L. Gary, T. Elaine Prewitt, Angela M. Odoms-Young, Joanne Banks-Wallace, Bettina M. Beech, Chanita Hughes-Halbert, Njeri Karanja, Kristie J. Lancaster, Carmen D. Samuel-Hodge

**Affiliations:** University of Pennsylvania School of Medicine, CCEB; Department of Epidemiology and Prevention, Division of Public Health Sciences, Wake Forest University Health Sciences, Winston–Salem, North Carolina; Department of Epidemiology, Johns Hopkins University Bloomberg School of Public Health, Baltimore, Maryland; Department of Health Policy and Management, Fay W. Boozman College of Public Health, University of Arkansas for Medical Sciences, Little Rock, Arkansas; Public Health and Health Education, School of Allied Health, College of Health and Human Sciences, Northern Illinois University, DeKalb, Illinois; School of Nursing and Program in Women and Gender Studies, University of Missouri, Columbia, Missouri; Department of Psychology, University of Memphis, Tennessee (current affiliation, Department of Internal Medicine and Public Health, Vanderbilt University, Nashville, Tennessee); Department of Psychiatry and Cancer Center, University of Pennsylvania School of Medicine, Philadelphia, Pennsylvania; Kaiser Permanente Center for Health Research, Portland, Oregon; Department of Nutrition, Food Studies, and Public Health, New York University, New York, New York; Nutrition Department, Schools of Public Health and Medicine, University of North Carolina at Chapel Hill, North Carolina

## Abstract

Obesity is more prevalent among African Americans and other racial and ethnic minority populations than among whites. The behaviors that determine weight status are embedded in the core social and cultural processes and environments of day-to-day life in these populations. Therefore, identifying effective, sustainable solutions to obesity requires an ecological model that is inclusive of relevant contextual variables. Race and ethnicity are potent stratification variables in U.S. society and strongly influence life contexts, including many aspects that relate to eating and physical activity behaviors. This article describes a synthesis initiated by the African American Collaborative Obesity Research Network (AACORN) to build and broaden the obesity research paradigm. The focus is on African Americans, but the expanded paradigm has broader implications and may apply to other populations of color. The synthesis involves both community and researcher perspectives, drawing on and integrating insights from an expanded set of knowledge domains to promote a deeper understanding of relevant contexts. To augment the traditional, biomedical focus on energy balance, the expanded paradigm includes insights from family sociology, literature, philosophy, transcultural psychology, marketing, economics, and studies of the built environment. We also emphasize the need for more attention to tensions that may affect African American or other researchers who identify or are identified as members of the communities they study. This expanded paradigm, for which development is ongoing, poses new challenges for researchers who focus on obesity and obesity-related health disparities but also promises discovery of new directions that can lead to new solutions.

## Introduction

Obesity is currently viewed as one of the most important health concerns in the United States and is an increasing focus of federally funded research (1,2). About 30% of Americans are classified as obese. Although overweight and obesity are problems for Americans overall, African Americans and other populations of color are disproportionately affected. Obesity prevalence data for African American women are especially alarming. Fewer than 20% of black women, compared with 33% of black men, have body weights in the range that is considered healthy (1). Nearly 15% of black women are in the "extremely obese" weight range, characterized by a body mass index greater than 40 kg/m^2^, equivalent to about 100 lb of excess weight. The prevalence of obesity among African American children is notably higher than among white children and has steeper trends (3). One-fourth of African American females aged 6 to 19 years are obese (1). Most African American women are trying to lose weight or control their weight, suggesting that the motivation for weight control is prevalent (4). However, knowledge of effective weight-loss approaches is derived primarily from white populations (5-7) and relates only to short-term effects. The need to tailor or adapt behavioral weight-loss approaches for specific populations is recognized, but effective ways to do this with African Americans have not been identified (8-11).

The purpose of this article is to describe a synthesis of efforts to build and broaden the research paradigm for obesity interventions in African Americans. Although at a basic level obesity results from an energy imbalance, (i.e., intake of excess calories in relation to caloric expenditure) behaviors such as overeating and physical inactivity that contribute to obesity are shaped by broader contexts, including social, economic, and political factors, and are embedded in the processes of day-to-day life (12-15). Consequently, research on effective approaches to obesity prevention and treatment must involve examination of complex pathways and ultimately must include identifying means of changing relevant contexts. Reliance on biomedical or individual behavior-change models, which are less inclusive of social and environmental variables, may limit the ability to identify effective, sustainable solutions. In such models, social contextual variables are often considered only indirectly, as barriers or facilitators to achieving targeted health goals.

Accordingly, identifying approaches to obesity prevention and treatment that are feasible and effective in African American communities requires a focus on life contexts in these communities. Work emanating from fields such as sociology and history has long supported the perspective that social and environmental contexts, particularly in the United States, are strongly defined by race and ethnicity (16).

## Synthesis of Broader Perspectives

The synthesis of efforts to build and broaden the research paradigm on obesity interventions in African Americans was initiated by the African American Collaborative Obesity Research Network (AACORN). The synthesis builds on the results of the organization's first national workshop, held in August 2004 in Atlanta, Georgia, in collaboration with the Centers for Disease Control and Prevention's Division of Nutrition and Physical Activity. The Appendix to this article includes a summary of key presentations and related references from the workshop. This workshop was consistent with AACORN's mission to stimulate research that will enhance the likelihood of effecting permanent solutions to obesity and obesity-related health problems in African American communities and will increase the visibility and agency of African American researchers in obesity research (17). Involving both community and researcher perspectives and drawing on and integrating insights from a broader set of knowledge domains expands the paradigm for obesity research to promote a deeper understanding of the relevant contexts.

In contrast to the typical focus on the health issue of obesity, in which aspects of the social context may be recognized only as they seem to interfere with individual behavioral change, the goal of this workshop was to *begin* with the social ecology (i.e., considering first the ways of life, assets, and challenges in African American communities) (18), and then attempting to determine where food, activity, and weight-related variables fit in. We argue that this procedure may give a more realistic picture of what anchors or motivates weight-related behaviors, what potential pathways for change are realistic and feasible, and what effective change will require. Although we are motivated by the particularly critical need to address obesity in African American communities, we believe that progression toward such an expanded paradigm may be needed in other ethnic minority populations, both generally and in relation to obesity.

## Reframing and Expanding the Research Paradigm: Concepts and Process

AACORN has articulated priority research recommendations (17) related to designing and conducting more contextually relevant and effective obesity prevention and treatment interventions. These research recommendations build on the National Institutes of Health (NIH) obesity research agenda (2,19), provide rationale and directions related specifically to African American communities, and include several recommendations that may improve the relevance of research on obesity prevention and treatment interventions by including the contextual variables of African American community and family life (17):

- Better characterize perceptions of food, eating, physical activity, and weight and their roles in African American family and community life.
- Determine the extent to which food, activity, and weight-related market behaviors of African Americans are influenced by targeted marketing and local availability of foods and how perceptions of buying power shape food choices.
- Obtain a better understanding of the processes whereby African American communities undergo cultural and structural changes and the key determinants of these processes.
- Design and evaluate weight-loss and weight-control strategies geared to African American families, social settings, and social dynamics.
- Increase qualitative and community-based research with African American communities to elicit underlying perceptions, issues, and priorities to derive insights for measurement and program design and to promote sustainability.
- Increase the number of African American researchers involved in obesity research, including researchers from disciplines such as family studies, child development, urban planning, and social work.
- Convene an ongoing, multidisciplinary group of experts, e.g., from health, education, human development, marketing, humanities, social science, economics, and urban planning, to develop comprehensive obesity research initiatives with African American communities.

These recommendations call for an increased emphasis on qualitative research and community-based participatory research (CBPR) and also for involving experts from a more diverse set of disciplines who can shed light on the "lifeworlds" of African Americans and provide a better sense of the community processes that need to be included in any framework for interventions. Lifeworld is a concept that emphasizes

. . . the centrality of perception for human experience. This experience is multi-dimensional and includes the experience of individual things and their contextual/perceptual fields, the embodied nature of perceiving consciousness, and the intersubjective nature of the world as it is perceived, especially our knowledge of other subjects, their actions and shared cultural structures (20). 

Consistent with the perspective described above, the paradigm expansion was guided by questions such as 

- Who can help us understand the deeper aspects of cultural and psychosocial processes that may influence the relationships of African American women to food?
- Who are the scholars who study aspects of day-to-day life in African American communities?
- What disciplines do these scholars represent? 
- Do they consider food and health issues in their work?
- How might their research approaches and methodologies differ from those typical in the obesity research field? 
- How can we access and integrate the knowledge and experience from these other fields? 

We also asked ourselves

- How do obesity research issues look from inside African American communities?
- What approaches can university researchers take to render research as relevant and effective as possible?
- How do approaches taken by African American researchers differ from those taken by other researchers?

The Figure describes the conceptualization and process involved in expanding the paradigm for obesity research with reference to African Americans. As the Figure illustrates, considerations related to the design and implementation of obesity-related interventions are viewed through three different lenses or perspectives: 1) those of African Americans in the communities of interest for research; 2) those of African American researchers; and 3) those of the research community in general, which also reflect the perspectives of research funders. Incorporating the views of community partners about obesity research and engaging community members through academic–community research partnerships is essential to this expanded paradigm. The perspectives of African American researchers are shown as interacting with community perspectives and linked to, but also distinct in some ways from, those of researchers in general (i.e., researchers who are not also members of the African American community). This emphasizes the importance of recognizing the dual perspectives that may apply when researchers are members (with respect to ethnic identity and shared history and experiences) of the communities they study and at the same time members of the academic community and accountable to research funders. While these dual perspectives play out for most researchers, many African American researchers, for the historical and sociopolitical reasons explained below, have to resolve intense social dichotomies inherent in this duality.

**Figure:**
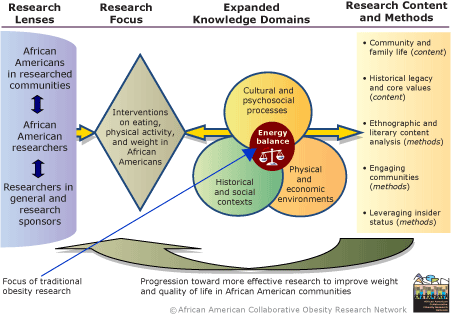
AACORN's Expanded Obesity Research Paradigm.

**Table:** 

Reprinted with permission of the African American Collaborative Obesity Research Network.

This paradigm expansion is based on the premise that the behaviors that determine weight status are embedded in the core social and cultural processes and environments of day-to-day life. Therefore, identifying effective solutions to obesity requires an ecological model that is inclusive of relevant contextual variables, which include variables influenced by race, ethnicity, and social position. *Center:* The traditional focus on caloric intake and output is depicted in the intersection of knowledge domains potentially informative for developing interventions on eating, physical activity, and weight. This representation conveys the utility of factoring in knowledge of historical and social contexts, cultural and psychosocial processes, and the physical and economic environments that influence preferences, perceived and actual choices related to food and activity, and the relative ease or difficulty of exercising these choices. Such knowledge is fundamental to understanding the perspectives and day to day experiences that are the backdrop for weight control efforts. Accessing relevant knowledge from these expanded domains is enhanced by interactions with scholars in fields such as family sociology, literature, philosophy, transcultural psychology, economics, marketing, and urban planning. *Left:* What is seen, asked, and heard depends on who is looking and listening. Important eyes and ears for understanding weight issues include those of lay members of the communities of interest (e.g., African Americans in researched communities) and researchers in relevant fields whose expertise incorporates insights based on lived experiences and shared identity with the community of interest (e.g., African American researchers in nutrition, physical activity, public health, and other areas), in addition to other researchers with relevant interests and expertise, and research sponsors. *Right:* Content and methodological themes emanating from this inclusive, integrative paradigm applied to African Americans highlight 1) the importance of considering family and community interactions related to food acquisition, food- and activity-related social interactions, the structure and organization of community processes, women’s roles, and differences by generation, social position, and other demographic variables; 2) the potential influence of the collective historical legacy of slavery and its derivatives on core values such as trust and loyalty and on interactions with the health care system, media, and other social institutions; 3) the potential value of qualitative investigations that include direct observations, eliciting and analyzing narratives, and exploring the content of literary expressions to yield different or richer insights than obtained from more typical biomedical approaches; 4) the essential need to fully incorporate the views, expertise, and agency of community partners in the research process; and 5) the potential benefits and challenges of encouraging African American researchers to leverage their insider status in ways that benefit the communities they study, the research endeavor, and their own academic careers.

As shown in the Figure, at the center of the process of expanding the research paradigm is the traditional focus on energy balance and weight control at the intersection of three knowledge domains that relate to African American communities ("expanded knowledge domains"). Historical and social contexts and cultural and psychosocial processes are fundamental aspects of African American lifeworlds as are physical and economic environments. The elements within each domain correspond to topics from family sociology, literature, philosophy, transcultural psychology, marketing, economics, and studies of the built environment (Appendix). Reflections on these knowledge domains from the three different perspectives ("research lenses") yield five interrelated themes that pertain to research content and methods. Content themes relate to the importance of community and family life and of the historical legacy and core values that shape the context for research in African American communities. Method themes relate to the potential value of qualitative approaches, such as ethnography and content analysis of literary works; engaging communities; and the ability of African American researchers to leverage their insider status in ways that benefit the research endeavor. Explanation of these themes is the main thrust of this article. The Table lists the subthemes identified within each theme. Cross-references to presentations in the Appendix are included, where applicable, to provide links to relevant references.

## Community and Family Life

Four subthemes emerged on community and family life contexts for obesity research (See summaries in Appendix for Robin Jarrett, Beverly Guy Sheftall, and Jerome Williams.)

### Community-specific environmental influences

African Americans are racially segregated and, as a population, are less likely than whites to live in physical environments that support healthful eating and physical activity. One quarter (24.7%) of African Americans live below the poverty line, and another 24% have incomes between 100% and 200% of the poverty line (21). The comparable figures for non-Hispanic whites are 8.6% and 15%. Hence, economic constraints on both food choices and opportunities for physically active recreation are disproportionate. Media and other marketing channels directed at African Americans facilitate the targeted advertising of products that are conducive to obesity (e.g., high-fat, low-nutrient foods). Yet these same channels can function as pathways for community-based interventions. Taken together, these environmental forces suggest above-average difficulty for African American adults in following recommendations for weight control for themselves or their children.

### Community structure and organization

Communities are complex systems with some layers that may not be visible to outsiders. Learning how communities are organized and how they operate may increase the ability to take a holistic or integrated approach to the design of programs to address food, activity, and weight. For example, how do families in low-income African American communities obtain their resources, and do they share them? What is the effective household income in an intergenerational household? What are the informal social networks that provide norm-setting, assistance, and support to people in the community? How do people take care of each other in times of difficulty, especially where major life challenges are constant phenomena?

Another aspect of structure and organization relates to temporal and spatial variation in how people interact within a neighborhood. Who controls what spaces? Is the same place or route safe at some times during the day but not at others? Ethnography suggests that identifying such sources of variation and understanding how people interact will give a different picture of life in communities than one would obtain on the basis of survey measures typically used to describe communities for comparative reasons.

### Women as a central focus

The high prevalence and severity of obesity among black women strongly influence the context for addressing obesity in African American communities. Many black women are heads of households. They have respected and influential roles in extended families, religious and civic organizations, and in other types of social networks, and they control most food shopping and preparation. Certain food-related roles may be integral to black women's positions of influence in their communities. Use of food as a mechanism to cope with stresses related to poverty, sexual abuse, violence, and racial discrimination is relatively more adaptive than coping through the use of alcohol or drugs as palliatives (22). Having a large body size may have adaptive elements as well (i.e., convey strength and power and offer protection from domestic and street violence). Researchers designing interventions to change eating behaviors or weight must be cognizant of the possibility that long-term changes in community perspectives related to food and weight could alter other aspects of the social ecology. In this sense it is impractical to design interventions on overweight or obesity that are incongruent with black women's needs and perspectives. Interventions may first need to address alternative ways to meet needs and evolve different or expanded perspectives on food, activity, and weight issues.

### Heterogeneity

The common misperception of African Americans or African American communities as homogeneous ignores within-group diversity. African Americans share common experiences in certain critical ways (e.g., the ongoing reality of racial discrimination, the history of slavery and related psychosocial and cultural legacies for the majority who are U.S.-born). Yet, the heterogeneity in African American communities also should be emphasized, for example, in differences in socioeconomic status, family types, neighborhood characteristics, and in urban versus rural areas and regions of the country; and diversity in values and psychosocial perspectives. Effects of socioeconomic status on obesity in African Americans are not always predictable based on patterns observed in whites. Aspects of being African American that are relatively independent of socioeconomic status may also influence obesity and related health problems.

## Historical Legacy and Core Values

The slave trade that brought people of African descent to the United States and its related history of inhumane treatment, coupled with contemporary circumstances of African Americans that evolved from this legacy, shape core values and norms within African American culture and deep psychosocial perspectives in African American communities. The following two subthemes require consideration. (See summaries in Appendix for Howard McGary and Linda James Meyers.)

### Historical importance of trust

Researchers should go far beyond the philosophical perspectives of trust in human relations to fully understand the concepts of trust and distrust in the setting of health disparities research and why it is so important to establish, gain, and maintain trust when working in African American communities. Survival during slavery, and especially during attempts to gain freedom, was highly dependent on blind trust and loyalty within networks and was coupled with ingrained distrust for anyone identified as benefiting from or maintaining slavery. This instinctive reliance on trust and distrust in guiding actions and alliances was reinforced during the 20th century civil rights struggle. It continues to be reinforced by various sociopolitical events and circumstances reflective of institutionalized racism, and it influences interactions of African Americans with the society at large. To permanently dispel distrust of health care institutions or their personnel among African Americans, for example, and to remedy the harm that can result from it will require that several generations of Americans fully embrace the principle that all people are, indeed, created equal — in other words, a major social change in the way that American society views its responsibilities to all citizens. Community-partnered research on obesity or other health issues can actually help to foster such social change. In any case, relying on superficial approaches or not understanding the reciprocal and long-term loyalty dimensions that come with gaining trust will be counterproductive.

### Collective trauma

The legacy of slavery and prolonged exposure to various forms of sustained socioeconomic and sociopolitical distress also has imposed intergenerational psychological consequences in the form of post-traumatic stress. This is analogous in concept to the legacy attributed to the Holocaust among Jews. The callous denial of humanity during slavery constitutes a collective trauma that has created a permanent yearning for a meaningful sociocultural grounding for African Americans. There are diverse individual experiences with, and expressions of, this need for grounding, both within and across generations. Individuals have different sensitivities to this trauma and a wide variety of ways of coping with it. The impact of this historical legacy on the collective of the African American community, therefore, varies with time, context, and situation.

Creative approaches are needed to uncover how these deeper issues of historical trauma can fit with the need for health promotion in African Americans generally and for weight control specifically. The continuing exposure to race-related adverse experiences influences the priority given to other aspects of the lifeworld. Combined with the effects of past and current exposure to material deprivation, community norms may condone behaviors such as overeating as acceptable coping strategies in spite of the effects on body weight. Collectively, African Americans' social concerns clearly supersede weight-related concerns, although weight-related concerns may add to the burden.

## Ethnography and Literary Content Analysis

Even well-validated survey instruments are often based on a general, superficial understanding of salient issues. That is, they may measure something well, but that "something" must always fit into preconceptualized pigeonholes. Particularly when used to study traditionally disenfranchised populations whose perspectives are not usually sought, naturalistic research methods can help researchers to gain insights that can only be obtained by "hearing the voices" of people inside these communities. Qualitative research requires personal commitment, lifetime involvement, and skills from the researcher that differ from those for quantitative research.

Two subthemes related to learning to hear the voices are the potential value of ethnography and the analysis of literary works. *(*See summaries for Robin Jarrett and Beverly Guy Sheftall in the Appendix.)

### Ethnographic research methods

Ethnography allows extended and repeated observations of people in their day-to-day life contexts and deepens the understanding of communities and their members (23). Ethnography can "unpack" the perceptions of reality by the groups under study and create a canvas of relevant information to guide intervention. Relevant to obesity, ethnography can identify unrecognized facets of community life that relate to eating, activity, and weight control.

### Literary representations of African American life

Content analysis of African American literary works to understand relevant issues in context is an unused or underused option for learning more about life in African American communities and understanding where and how food and weight issues fit in. Referring to works of African American writers or film producers, for example, takes advantage of the insights of these artists in identifying truths that reflect variations on the collective, lived experiences of African Americans. In addition to the potential value of this approach for gaining a better understanding of the context for weight-related interventions, literary works, film, and television productions may also provide innovative vehicles for interventions (i.e., health promotion messages could be incorporated into these works).

## Engaging Communities

The research lenses in the Figure remind us of the differences in research perspectives of community members and academic researchers and of the need for CBPR to integrate and balance these perspectives (24). Although the importance of genuine CBPR approaches is clear, the potential benefits of CBPR to communities and their members may be compromised by unintended consequences of poor implementation, which means that the research itself may also be compromised (25). It is essential to clarify how best to achieve community involvement in a manner that is mutually satisfactory and beneficial to community and academic partners. This means, foremost, the creation of trust and of a social contract of long-term loyalty. It implies explicit steps for creating opportunities for communities to discuss their perceptions of health problems, what resources they have, what priorities they set, and to determine their sense of what approaches have the potential to remedy problems.

The differences in perspective, the social distance, and the power inequity between academic researchers and community-based research partners are major hindrances to increasing mutual trust and respect (25). Community-based research partners are in a relatively weak position with respect to obtaining funding directly, which renders them dependent on and subordinate to universities, which further complicates their relationship with academic researchers. (See summaries in Appendix for Margaret Grayson and Anna Huff, Robin Jarrett, Howard McGary, and Linda James Meyers.)

### Community members as equal research partners

Academic institutions and researchers have traditionally viewed researched communities as "subjects" (i.e., people and settings that researchers can probe and rectify with their expertise). It is difficult to change this cultural lens of the professional researcher; a new lens is needed. Researchers must view community partners as equals who bring vital and different knowledge and expertise to the research process. Without this view, changes in interactions with community partners may be implemented on the surface but not carried through on other levels (e.g., related to decision making and sharing of resources), even by those who are well-intended and who embrace, intellectually, the need for CBPR. Insufficient commitment to this aspect of equity will create a backlash because of the aforementioned problems of trust and distrust. Learning and training should be bidirectional between academic researchers and community partners and should involve a commitment to hearing and speaking truth from both directions.

### Community strengths

Partnering with community members and organizations helps academicians to learn from community members' knowledge and experience. Partnering will also identify strengths and opportunities often overlooked that can be leveraged for health improvement. Communities that survive under difficult economic and sociopolitical conditions have, by definition, developed ways to work around or remove barriers and live with or solve complex problems. The survival skills of community members, when properly understood, may also be potential assets that can facilitate both participation in health research and improvements in community health. Health research participation and improved community health can, in turn, add to community capacity and community strengths.

### Increase direct benefits to researched communities

Communities benefit more from research if resources are shared, thereby avoiding duplication and the attendant potential for excessive burden, and also facilitating synergistic effects among projects and over time. Our paradigm fosters coordination among researchers studying the same communities and feedback to communities about the results of research conducted in their or similar communities.

## Leveraging Insider Status

The research lenses in the Figure highlight the dual perspectives that may occur when African American researchers identify or are identified as members of the communities they study while they also maintain allegiance and accountability to the academic research enterprise. African Americans are connected by a shared social history. Researchers function in a professional context and culture. Hence, this dual perspective is unavoidable. When the duality relates to an ethnic identity that is associated with a long history of denigration, abuse, and denial of humanity, it becomes potentially distressing. Even when African American researchers who, for reasons of social class or lived experience, do not identify personally with African American research participants, they may nevertheless be viewed as "one of us" by study participants or community members because they are African American. This scenario creates subtle and not so subtle challenges, both in the academic community and in the ethnic community (26). There are several important, interrelated subthemes:

### Establishing trust and credibility

Insider status may create the potential for greater rapport and trust, but this is not automatic. African American health professionals and researchers may encounter distrust or low credibility if they fail to acknowledge their commonality with some of the same issues faced by the people from the community. Finding an effective social distance or posture may be difficult, and what is effective in this respect may change over the course of relationships with communities. On the other hand, appearing to overidentify (i.e., with one's ethnic community) may lead to alienation from peers in the scientific community and role confusion for the researcher. African American researchers conducting research with African American communities may have an almost daily dilemma in how to find and maintain trust and credibility in both worlds.

### Connections to communities of reference

Research in African American communities that is conducted by African Americans will still involve cross-cultural relationships in certain respects (i.e., professional perspective, social class, or other experience-based variables). Cross-cultural relations require constant attention to the double lenses that are used and take a long time to fit comfortably (27). In principle, in using CBPR, the connection with the community becomes open-ended and will flow from research to service, to advocacy, back to research, to social relations, and then to more research. For many African American and other ethnic minority researchers, retaining ongoing, close, personal connections with communities of reference may be critical to assessing and enhancing insider knowledge and evaluating the validity of professional perspectives. This process also provides opportunities to reciprocate with community service, where that is desirable, and to renew the motivation to make the effort needed to conduct research under complex and difficult circumstances. However, remaining connected also means continuing to experience — at least through empathy — the problems one is trying to address or to work around. Conducting research as an insider is, therefore, also potentially more stressful than when one can maintain an outsider perspective. One may hear the voices long after the data have been analyzed and the paper published.

### Objectivity and expectations

Researchers who have advocacy goals or insider perspectives may feel, and perhaps want, to be personally involved with and affected by the research participants and the subject matter. This desire for personal involvement goes against the grain of the prevailing biomedical paradigm, which stresses maximum objectivity of researchers and in which a relatively detached stance toward research participants is the norm. In addition, identification with the researched community or being identified by the community as an advocate may also create demands (external or self-imposed) to ensure that the community benefits in some immediate way from providing access and participation. The business of research has potentially exploitative elements. The desire or obligation to give back to the community may be a major consideration for some researchers but may not be highly valued or rewarded by the academic institution sponsoring the research. Strong tensions develop between institutional demands or practices and a researcher's commitment to the well-being of the community in which the research is focused or based, although this tension varies among institutions. The greatest problems may arise when the institution in question is located in or very close to the communities where the research takes place and where historic academic–community relationships have been difficult, as is true for many U.S. academic medical centers. Nevertheless, the desire to see communities benefit is a legitimate and ethical consideration for how researchers interact with communities and how they view the balance of benefits to communities and institutions. Ideally, researchers can provide service to the communities in ways that also stimulate and sustain community members' interest in, and capacity for, participation in health research.

### Finding social and professional support

Researchers as well as mentors and colleagues should acknowledge the psychological and social demands associated with functioning in a combined insider–outsider role and should appreciate the potentially above-average or qualitatively different needs for supportive environments. Both the time and the opportunities to obtain appropriate and adequate support are needed.

## Implications

No consensus exists on how to improve obesity research in communities of color (8-10). (See summary in Appendix for Shiriki Kumanyika.) Various types of cultural adaptations have been used in studies with African Americans: limiting the audience (inviting only African Americans to participate); matching staff ethnicity by hiring only African American staff; including content designed to appeal to the perceived sociocultural perspectives of African American audiences; and including community members in the conceptualization and development of program content and format and to access traditions, views, norms, core values, and language or culturally influenced perspectives (8,9).

These types of adaptations apparently do not confer a dramatic advantage in the ability of African Americans to lose weight (8). Many approaches used are intuitively appealing and theoretically based to some extent (8,9), but culturally adapted programs have often yielded disappointing results. Weight losses reported in published studies of programs that include such adaptations have often been negligible or very small, and retention rates are not necessarily better than in other programs (8,10). The reasons for low success rates in studies of culturally adapted programs — in which success is judged on the basis of attained weight loss — may be explained by structural differences between these types of studies and clinical studies of efficacy that yield more impressive weight losses. Culturally adapted programs differ from efficacy trials in that their settings are less amenable to control by the researcher; their duration is often shorter; the study populations are more heterogeneous and less likely to have been selected on the basis of their ability to adhere to the program; behavior change approaches are less strict and are designed to be more responsive to participant needs and interests; and the staff are less likely to be highly trained specialists. Although these characteristics often render culturally adapted programs more relevant to and representative of the community of interest and more feasible for dissemination, they also tend to reduce the strength or effective dose of the weight-loss counseling delivered.

On the other hand, the possibility that other important outcomes are positively affected by such programs should be considered. Weight control programs touch upon many aspects of people's lives other than specific eating and physical activity behaviors. For example, behavior-change counseling may target various cognitive processes, coping strategies, stress management, social skills, and personal administrative skills, such as time management, priority setting, and problem solving, and may encourage self-reflection and building of self-confidence. Benefits in these areas may occur whether or not the weight-loss goal is achieved during the applicable follow-up period and could ultimately have positive implications for the health or quality of life of participants. Assessing such benefits could provide important insights about the dynamics of culturally adapted programs.

Particularly pertinent is our assessment that the emphasis on *culture* in studies conducted to date has been relatively narrow and has focused on sociocultural attitudes and beliefs about food or physical activity or on changing the intervention setting without attention to the more fundamental social context issues we have explored here. We have identified few whole-community or comprehensive studies relevant to obesity prevention or control (11). As we have discussed, individual behavior issues related to eating and physical activity are intertwined with social, physical, economic, and policy environments. Some very fundamental and structural aspects of the environments of African American life are biased toward unhealthy dietary patterns and physical inactivity (28,29). (See presentations by Jerome Williams, Adam Drewnowski, and Kristie Lancaster and Melicia Whitt-Glover in Appendix.) Weight gain may be incidental to coping strategies such as an overreliance on food to manage emotions, cope with stress, or reward children, combined with reliance on television watching for recreation and to keep children engaged because it is affordable and safe. Individually oriented interventions that address cultural attitudes and beliefs out of context will be insufficient to alter patterns of day-to-day living that are conditioned responses to environmental circumstances. In fact, the entire approach to adapting obesity prevention interventions to the "culture" of African American communities should be reexamined and reframed to be consistent with the expanded paradigm proposed here, that is, based on an ecological model that leads to environmental and policy approaches and also addresses relevant themes in family and community life.

## Conclusion

AACORN's 2004 workshop initiated a process of "out-of-the-box" thinking about how to frame and study obesity issues in African American communities. The themes identified from this process add structure and detail to the previously developed AACORN research agenda (17), particularly with respect to the importance of multidisciplinary expertise, qualitative research, and high-quality CBPR. The results of this synthesis suggest the type of research that the authors, as African American scholars in fields related to nutrition, physical activity, and obesity, feel especially suited to pursue given our lived experiences and accumulated knowledge. The expanded paradigm highlights the need for African American researchers to become more numerous, effective, and acknowledged in the obesity research field. However, it also highlights the challenges faced by researchers who are linked, through ethnic identity and shared experiences, to the communities they study while also functioning in academic contexts that may not value either the communities or community-based research.

The expanded paradigm described here suggests that weight-control interventions must be framed more holistically to consider other relevant social and health priorities and to allow for the possibility that excess weight is part of a complex set of adaptations to adverse life circumstances (30). This paradigm, for which development will be ongoing, means new challenges for researchers who focus on obesity and obesity-related health disparities and promises discovery of new directions that can lead to new solutions. We are convinced of the potential value of casting the net widely to identify and work with disciplines and scholars who can contribute to this line of research. Along these lines, the second national AACORN workshop held in August 2006 further explored some of the themes discussed here in a dialogue with researchers and community partners whose work addresses critical contextual issues such as violence prevention, high prevalence of incarceration, housing needs, and environmental justice issues (31).

## Figures and Tables

**Table.** Obesity-Related Research Content Areas, Issues, and Approaches Identified Through Synthesis of Workshop Presentations and Discussion, Second Annual Scientific Meeting and Workshop, Achieving Healthy Weight in African American Communities: Interdisciplinary Research Directions, 2004

Theme	Subthemes
Community and family life	Community-specific environmental influences Community structure and organization Women as a central focus Heterogeneity
Historical legacy and core values	Historical importance of trust Collective trauma
Ethnographic and literary content analysis	Ethnographic research methods Literary representations of African American life
Engaging communities	Community members as equal research partners Community strengths Benefits to researched communities
Leveraging insider status	Trust and credibility Connections to communities of reference Objectivity and expectations Social and professional support

## Appendix

### AFRICAN AMERICAN COLLABORATIVE OBESITY RESEARCH NETWORK

*Second Annual Scientific Meeting and Workshop*
Achieving Healthy Weight in African American Communities: Interdisciplinary Research Directions
Sponsored by the Division of Nutrition and Physical Activity, Centers for Disease Control and Prevention
August 9–10, 2004
Wyndham Hotel Downtown, Atlanta

Reprinted with permission of the African American Collaborative Obesity Research Network.

### Selected Workshop Presentation Summaries

#### BACKGROUND

In August 2004, the African American Collaborative Obesity Research Network (AACORN) convened its first interdisciplinary workshop in collaboration with the Centers for Disease Control and Prevention's (CDC's) Division of Nutrition and Physical Activity.

The objective was to explore ways to break new ground in developing effective approaches to address the high prevalence of obesity and related health problems in African American communities. Workshop participants included university researchers; research trainees; community partners engaged in food intake, physical activity, or obesity research or programs in African American communities; and several CDC staff.

Presenters included scholars from fields not traditionally identified with obesity research: family sociology, literature, philosophy, and transcultural psychology. Community partners presented their perspectives on academic–community research partnerships. Other presenters addressed media and marketing issues, food economics, built environment effects on food intake and physical activity, and obesity prevention and treatment studies. Overall, the workshop planted the seeds for research bridging a broad spectrum of disciplines to challenge the primarily biomedical paradigm still used to study obesity, food intake, and physical activity. Conclusions pointed to potential directions for researchers and community partners who study these topics — particularly those who focus on health disparities.

#### CONTENTS

**Session 1. Historical, Cultural, Sociological, and Literary Influences on Food, Activity, and Weight**

*Contemporary Sociological Factors Influencing African American Life*
Robin Jarrett, PhD

*Expressions of African American Life in Literary Works*
Beverly Guy Sheftall, PhD

*Philosophical Underpinnings of African American Cultural Values*
Howard McGary, PhD

*The Collective Psyche of African Americans*
Linda James Myers, PhD

**Session 2. Environmental Influences on Food, Activity, and Weight**

*Influence of Media and Marketing*
Jerome Williams, PhD

*Impact of the Economy/Economics*
Adam Drewnowski, PhD

*Impact of the Built Environment on Food Intake and Physical Activity*
Kristie Lancaster, PhD, and Melicia Whitt-Glover, PhD

**Session 3. Where Are We Starting?**

*Current Status of the Literature on Obesity Prevention and Treatment in African Americans*
Shiriki Kumanyika, PhD, MPH

**Session 4. Community Perspectives**

*Southeast Missouri Community Partners for Progress, Inc., and Mid-Delta Community Consortium of the Arkansas Rural Development Network*
Margaret Grayson and Anna Huff

### Contemporary Sociological Factors Influencing African American Life

Robin Jarrett, PhD
Associate Professor, Human and Community Development
University of Illinois at Urbana-Champaign
Urbana, Illinois

Dr Jarrett provided an in-depth perspective of African American families living in low-income communities in Chicago. She emphasized the importance of using ethnographic methods, such as life histories, to understand the complexities of African American family life; families defined not only by kinship and marriage but also by other interpersonal, sustained family-like relationships. Such relationships provide the basic survival network (e.g., for acquiring resources for food and recreation). Dr Jarrett highlighted the sub-cultural issue of very protective, dominant mother roles, where these women play a central role in resource provision and management. She discussed differences in structure and functioning of "female-headed" and "extended family households"; ways that families pool resources and cooperate; movement in and out of households depending on circumstances, including life transitions; within-household family dynamics, such as cross-generational and collective parenting; the significance of social roles in defining women's identity and self-worth; and the diverse sources of income, including the role of youth wages and "underground economies."

Dr Jarrett also offered insights about families' perceptions of "good places" and "bad places" within neighborhoods, times of day considered safe or unsafe to be outside, and perceptions of community assets and needs. The family processes and neighborhood contexts provide the multifaceted reality in which health interventions are developed and implemented. This reality pertains to priority setting, role assessments, decisions and responsibilities for food acquisition and preparation, broad and dynamic family structures that downplay individualism, as well as time and place assessment and management.

**Related Readings**

1. Jarrett RL, Jefferson SM. Women's danger management strategies in a public housing project. Fam Rel 2004;53:136-147.
2. Jarrett RL, Jefferson SM. A good mother got to fight for her kids: maternal management strategies in an urban housing project. J Chil Pov 2003;9:21-39.
3. Jarrett RL. Neighborhood effects models: A view from the neighborhood. Research in Community Sociology 2000;10:305-323.
4. Jarrett RL. Voices from below: the use of ethnographic research for informing public policy. In: Mercier JM, Garasky S, Shelley MC, editors. Redefining family policy: implications for the 21st century. Iowa State University Press; 2000. p. 67-84.
5. Jarrett RL, Burton LM. Dynamic dimensions of family structure in low income African American families: Emergent themes in qualitative research. J Comp Fam Stud 1999;30:177-187.
6. Jarrett RL. Successful parenting in high-risk neighborhoods. Fut Child 1999;9(2):45-50.

### Expressions of African American Life in Literary Works

Beverly Guy Sheftall, PhD
Anna Julia Cooper Professor of Women's Studies and English
Director, Women's Research and Resource Center
Spelman College
Atlanta, Georgia

Dr Sheftall explored literary works by or about African Americans to derive insights about eating, food, and weight issues. Most texts stressed the importance of a multilayered approach to the topic, acknowledging the intersection of race, class, gender, and sexuality. She emphasized the need for solutions attentive to the complexities and heterogeneity of the "black experience," particularly the need for different approaches to reach the young generation. A gender-specific approach is crucial, and she suggested the need to confront taboo subjects such as sexual abuse, which may contribute to women's food and weight issues. Dr Sheftall stated that African American feminist writers have argued the salience of chronic overeating to suppress emotions, particularly around incest, rape, domestic violence, and sexual abuse. Other reasons for overeating among black women relate generally to racism, including those implicit in prevailing white- and thinness-oriented standards of beauty. Complex issues surrounding body image, appetites, dietary practices, food obsessions, emotional deprivations, and self-esteem related to physical appearance were readily identifiable in literature by and about black women. A clear message in the writings of African American women is a dearth of self-love. We need to better understand how this relates to unhealthy or seemingly self-destructive behaviors, including eating habits, food addiction, and food as a substitute for love. In terms of self-identity, Dr Sheftall argued that in literary works the social context for African American women includes dominant portraits of roles as cooks, for their own and others' families, making it nearly impossible to disconnect the notion of African American women from food. In fact, the variables that perpetuate overeating in black women are so entrenched that effectively addressing obesity requires profound social transformations.

**Related Readings**

***African American Writers***

1. Brown R. Full-figured women fight back. Ebony 1990 March; 27-31.
2. Ferrell C. Eating confessions. Callaloo 1989;12(3):453-64.
3. Hooks B. Sisters of the yam: black women and self-recovery. Boston: South End Press; 1993.
4. Jordan J. Free flight. In: Passion: new poems, 1977-1980. Boston: New Press; 1980.
5. Naylor G. Linden Hills. New York: Viking, 1985.
6. Powers R. Fat is a black woman's issue. Essence 1989 October; 75, 78, 134, 136.
7. Walker A. Meridian. New York: Simon & Schuster, 1976.
8. Avery B. Breathing life into ourselves: the evolution of the national black women's health project. Arnold G. Coming home: one black woman's journey to health and fitness. In: White E, editor. Black women's health book: speaking for ourselves. Seattle: Seal Press; 1990.
9. Witt D. What (n)ever happened to Aunt Jemima: eating disorders, fetal rights, and black female appetite in contemporary American culture. In: Wallace-Sanders K, editor. Skin deep: spirit strong: the black female body in American culture. Ann Arbor: University of Michigan Press; 2002.

****Other Texts****

1. Bordo S. Unbearable weight: feminism, western culture, and the body. Berkeley: University of California Press; 1993.
2. Orbach S. Fat is a feminist issue: a self-help guide for compulsive eaters. New York: Berkeley-Paddington; 1978.
3. Thompson BW. A hunger so wide and deep: a multiracial view of women's eating problems. Minneapolis: University of Minnesota Press; 1994.
4. A way out of no way: eating problems among African-American, Latina, and white women. Gndr & Soc 1992;6(4):545-61.
5. Yancey A. Ain't like there's hunger. Kent: Imhotep Publishing, Inc; 2003. (Also published in Am J Prev Med. 2003;25[3Si]:1.)

### Philosophical Underpinnings of African American Cultural Values

Howard McGary, PhD
Professor of Philosophy
Rutgers University
New Brunswick, New Jersey

Dr McGary offered a normative approach for considering obesity, involving the aesthetic, moral, religious, and social values in African American communities. He focused on loyalty, justice, and trust as they pertain to African American communities.  Dr McGary traced these values primarily to ideas and traditions transmitted in families from slavery onward. For example, loyalty had important empirical consequences foremost for keeping family together and ensuring that the family was safe and protected. In reviewing the importance of justice,  Dr McGary indicated that African Americans have traditionally linked justice to their struggle for freedom from slavery and, later, for civil rights. African Americans have therefore always viewed justice in terms of the rights of individuals *and* of groups, along the lines of political philosopher John Rawls. This view is different from the "consequentialist" approach of John Stuart Mills, which views just actions as those that bring the greatest happiness to the greatest number of people in society. Similarly, the importance of trustworthiness was linked to the need for people in politically oppressive or socially disadvantaged situations to be able to identify individuals or institutions on whom they could rely for protection from significant bodily, emotional, or economic harm. Against this background, Dr McGary explored the origins of mistrust of institutions by African Americans and paid special attention to the health care system and its professionals. He discussed the historical erosion of trust that African Americans experienced in the hands of the health care system during and long after slavery was abolished. This might explain why African Americans may fail to heed what many rational people would consider sound advice about adopting behaviors that could curb obesity.  Dr McGary argued that the U.S. health care system is obliged to regain trustworthiness among clientele such as African Americans who might have legitimate reasons for distrust. This obligation is separate from the equally important duties of health care systems to treat everybody, including African Americans, fairly.

**Related Readings**

1. McGary H. Race and social justice. Malden: Blackwell Publishers; 1998.
2. Racial groups, distrust, and the distribution of health care. In: Rhodes R, Battin M, Silvers A, editors. Medicine and social justice. London: Oxford University Press: 2002. p. 212-223.
3. Distrust, distributive justice, and health care. Mt Sinai J Med 1999;66(4):236-240.
4. McGary H. Distrust, Social justice, and health care. Mt Sinai J Med 1999;66(4):236-40.

### The Collective Psyche of African Americans

Linda James Myers, PhD
Associate Professor, African American and African Studies and Psychology
Ohio State University
Columbus, Ohio

Dr Myers discussed the importance of culture, particularly cultural deep structure, the uniqueness of the experience of African American people, and approaches to achieving optimal health in African Americans. Describing African American cultural assets and African cultural traditions and values, Dr Myers explored the need to overcome multi-generational experiences that have contributed to psychological trauma and "menticide" (defined as the systematic destruction of a people's mind and culture). Her work develops a corrective "optimization process" to help African Americans regain a cultural grounding, consequently enabling them to understand their intrinsic worth. This process directly opposes the socialization African Americans receive in the dominant American culture that indoctrinates human beings into valuing external criteria — how they look, where they live, their income, and their position. Dr Myers also posited the possibility of *positive* outcomes from long-term adversity among African Americans, including the development of highly refined capacities for critically self-reflective and introspective thinking regarding coping in American society and substantial internal fortitude and resilience to survive, and in some cases flourish, under extremely disadvantageous circumstances. These qualities could be leveraged in favor of optimizing psychological and physical health and well-being in African American communities.

**Related Readings**

1. Myers LJ. Understanding an Afrocentric worldview. Introduction to an optimal psychology. 1993. www.optimalpyschology.net
2. Myers LJ. The deep structure of culture. Relevance of traditional African culture in contemporary African life. In: Hamlet JD, editor. Afrocentric visions studies in culture and communication. Thousand Oaks (CA): Sage; 1999. p. 3-14.
3. Montgomery DE, Fine MA,  James Myers L. The development and validation of an instrument to assess an optimal Afrocentric world view. J Blk Psych. Special Issue: Incorporating an African world view into psychology. 1990:17(1):37-54.
4. Fine MA, Schwebel AI, James Myers L. Family stability in Black families: Values underlying three different perspectives. J Comp Fam Stud 1987; 18(1):1-23. 
5. Constantine MG, Donnely PC, James Myers L. Collective self-esteem and Africultural coping styles in African American adolescents. J Blk Stud 2002;32(6):698-710.

### Influence of Media and Marketing

Jerome Williams, PhD
F. J. Heyne Centennial Professor in Communication
The University of Texas at Austin
Austin, Texas

Any effort to address determinants of obesity will encounter marketing issues. Dr Williams clarified his perspective as one of helping people outside marketing understand how marketers think. His data and graphical presentation of advertisements strongly suggested that consumption and marketing of soft drinks and less healthful foods are important foci for research on weight control among African Americans. His investigative methods include market research designed to study consumption patterns, use of supermarket scanner data to identify food purchasing patterns, and content analysis of advertising, television shows, and movies. He emphasized that trying to convince marketers to use different, more health-conscious principles is a daunting challenge. One encounters the "chicken and egg" problem when trying to assign responsibility to marketers for adverse food and beverage consumption patterns. Companies assert that they market in ways that provide what consumers want, as a basic tenet of profit making. Public health professionals may attempt to show that healthier consumers are more profitable than unhealthy ones and that for marketers to help shape consumer preferences by creatively and aggressively marketing healthier product lines is good for business. Dr Williams felt that the most fruitful approach would be trying to understand and change African Americans' attitudes about what is in their best interests, to influence demand curves so that marketers would have to react accordingly. However, he acknowledged that commercial markets have far more resources at their disposal to perpetuate current practices than do public health professionals to shift demand variables. He cited market research indicating that African Americans and Latinos consume carbonated beverages (regular soft drinks; data for diet soft drinks show underconsumption by African Americans and Latinos relative to whites) at significantly greater rates than the general population and in certain geographic market areas account for 60% to 70% of demand. Hence, it is not surprising that there are proportionately more soft-drink advertisements on television shows that have a high African American audience. The same reasoning and types of evidence are available for food more generally, including fast food. Furthermore, in addition to commercial promotions of various foods in conventional form, the balance of foods portrayed in movies does not coincide at all with dietary recommendations. African Americans portrayed in television shows and movies geared to black audiences seem heavier on average. These lines of research are at an embryonic stage but should be pursued more substantively.

**Related Readings**

1. Moore DJ, Williams JD, Qualls WJ. Target marketing of tobacco and alcohol-related products to ethnic minority groups in the United States. Ethn Dis 1996 Winter-Spring;6(1-2):83-98.
2. Williams JD, Tharp MC. African Americans: ethnic roots, cultural diversity. In: Tharp MC. Marketing and consumer identity in multicultural America. Thousand Oaks (CA): Sage; 2001.
3. Williams JD, Lee WN, Haughtvelt CP. Diversity in advertising: broadening the scope of research directions. Mahwah (NJ): Laurence Erlbaum Assocs; 2004.
4. Harris AMG, Henderson GR, Williams JD. Courting customers: assessing consumer racial profiling and other marketplace discrimination. J Pub Pol Mktg 2005;24:163-171.
5. Grier SA, Brumbaugh AM. Noticing cultural differences: ad meanings created by target and non-target markets. J Advertising 1999;Spring;79-93.
6. Aaker JL, Brumbaugh AM, Grier SA. Nontarget markets and viewer distinctiveness: the impact of target marketing on advertising. J Consum Psychol 2000;9:127-40.
7. Tirodkar MA, Jain A. Food messages on African American television shows. Am J Public Hlth 2003;93(3):439-41.
8. Henderson VR, Kelly B. Food Advertising in the age of obesity: content analysis of food advertising on general market and African American television. J Nutr Ed Behv 2005;37(4):191-96.
9. Williams, JD, Kumanyika S. Is social marketing an effective tool to reduce health disparities? Soc Mar Q 2002;VIII(4):14-31.

### Impact of the Economy/Economics

Adam Drewnowski, PhD
Professor, Epidemiology and Medicine
Director, Food Intake Science Program
University of Washington
Seattle, Washington

Food economics — the relative price structure of different types of food — influence food intake and weight and put people in low-income communities at a disadvantage with respect to food purchases. Dr Adam Drewnowski demonstrated that income limitations influence the sources of calories, or the types of foods people buy. Contrary to the longstanding view that healthy diets were merely a matter of personal responsibility, it is now recognized that individual food choices are adversely affected when the environment offers abundant inexpensive, calorically dense foods that are convenient and are good tasting, but unhealthy — although whether one can actually consume a healthful diet at low cost continues to be controversial.  Dr Drewnowski's analyses show that dietary quality is higher and obesity prevalence lower at higher levels of various indices of income or wealth. The link between socioeconomic status and obesity may, therefore, be mediated by factors such as unequal access to healthy diets. As resources diminish and people minimize food costs, they may substitute less expensive for more expensive foods within a given category to maintain their caloric intake: canned tuna for salmon; bologna for steak; apples for strawberries. When resources diminish still further, certain foods may be abandoned because they are simply too expensive. What remains are refined grains, added sugars, and added fats, because they are the cheapest foods. Economic and physiological factors work together to promote overeating in that the cheapest foods are also high-fat or high-sugar foods that have an innate appeal to the human appetite. These foods may also have less satiating power and are, therefore, too easily overeaten. This leads to a paradox in which people who are trying to save money on food actually end up eating more.

Dr Drewnowski noted that these economic variables are linked to agricultural policies such as food subsidies that depress the cost of fat and sugar and, therefore, the cost of high-fat, high-sugar foods. The Thrifty Food Plan — the market basket model that determines minimum income needed to support an adequate diet — is another policy for which the underlying assumptions are probably unrealistic and simplistic with respect to how low-income families may function (e.g., the time or willingness to prepare foods from more affordable basic ingredients, the spending priorities and cycles of low-income households [e.g., spending more when Food Stamp funds are received and then cutting back later in the month]).

Noting that economic factors include marketing patterns that result in a dearth of supermarkets and surfeit of high-fat and high-sugar foods in low-income and minority communities, Dr Drewnowski advocated for research related to neighborhood food environments, although with a much broader perspective and finer level of detail than has been the case to date. Local economic issues such as how food production, retail marketing, and restaurant industries are linked to the livelihoods of African American communities and families should be considered. Other issues include the type and pricing of foods in different neighborhoods relative to purchasing power, attitudes, and preferences of consumers (e.g., about what constitutes a healthful diet, brand preferences or perceptions that link certain food types to social status, healthfulness), implications of various policies that might be instituted, and food quality and safety issues. Studies might also investigate how changes in economic status (e.g., linked to community development programs) influence changes in what types of foods are available and purchased, and whether increased income leads to improved eating patterns without the addition of food intake-specific interventions.

**Related Readings**

1. Drewnowski A, Darmon N. The economics of obesity: dietary energy density and energy cost. Am J Clin Nutr 2005;82(1 Suppl):265S-273S.
2. Drewnowski A, Darmon N. Food choices and diet costs: an economic analysis. J Nutr 2005;135(4):900-4.
3. Drewnowski A. Obesity and the food environment: dietary energy density and diet costs. Am J Prev Med 2004;27(3 Suppl):154-62. 
4. Drewnowski A, Darmon N, Briend A. Replacing fats and sweets with vegetables and fruits — a question of cost. Am J Public Health 2004;94:1555-9. 
5. Drewnowski A, Specter SE. Poverty and obesity: the role of energy density and energy costs. Am J Clin Nutr 2004;79:6-16.
6. Karp, RJ, Cheng C, Meyers AF. The appearance of discretionary income: influence on the prevalence of under- and over-food intake. Int J Equity Health 2005;4:10.

### Impact of the Built Environment on Food Intake and Physical Activity

Kristie Lancaster, PhD
Assistant Professor, Department of Food Intake and Food Studies
New York University
New York, New York

Melicia Whitt-Glover, PhD
Assistant Professor, Department of Public Health Sciences
Wake Forest University School of Medicine
Winston–Salem, North Carolina

Drs Kristie Lancaster and Melicia Whitt-Glover summarized current knowledge and potential future research directions related to built environment issues (i.e., buildings, transportation systems, community design with respect to land use, locations of businesses), as they inform the potential for achieving healthful weight in African American communities. Built environment issues are now a major focus of obesity research as the context that determines what options, challenges, and barriers exist for making and reinforcing food intake and physical activity choices.

Dr Lancaster discussed built environmental factors that may contribute to lower intake of recommended foods and nutrients among African Americans. She cited data indicating an association between a high prevalence of obesity in New York City neighborhoods and low reported fruit and vegetable intakes in those neighborhoods. Dr Lancaster collaborated on a study in which the availability in grocery stores of five foods recommended for people with diabetes was compared between a high income, primarily white neighborhood (New York City's Upper East Side) and the adjacent, low-income and primarily African American and Latino neighborhood (East Harlem). Their findings showed that East Harlem stores were significantly less likely to carry all five of the recommended items. A few studies, all except one of those that are relevant, have reported a positive association between availability of healthy foods in neighborhood stores and intake of those foods by people who lived in the neighborhood.

Dr Whitt-Glover reviewed evidence on how the ability to engage in physical activity may be influenced by factors in the physical environment. Aspects of the natural environment (e.g., hills, weather, scenery) clearly influence outdoor activity. However, built environments are also influential through many variables common in urban, inner city communities: heavy traffic, lack of street lights, high crime, or other threats to personal safety, although urban areas are generally more walkable in other respects. Higher levels of physical activity are associated with variables more common in suburban areas and areas with smaller black populations: access to facilities (both perceived and actual) for physical activity/exercise, satisfaction with facilities, presence of sidewalks/foot paths (e.g., parks, green spaces, bike paths and lanes, mixed land use [e.g., a mix of residential and commercial destinations]). Several studies have noted that of all environmental concerns, lack of safety as a deterrent to engaging in physical activity, particularly walking, was most salient among black women compared to women of other racial and ethnic groups.

**Related Readings**

1. Edmonds J, Baranowski T, Baranowski J, Cullen KW, Myres D. Ecological and socioeconomic correlates of fruit, juice, and vegetable consumption among African-American boys. Prev Med 2001;32(6):476-81.
2. Eyler AA, Matson-Koffman D, Young DR, Wilcox S, Wilbur J, Thompson JL, et al. Quantitative study of correlates of physical activity in women from diverse racial/ethnic groups: The Women's Cardiovascular Health Network Project — summary and conclusions. Am J Prev Med 2003;25(3 Suppl 1):93-103.
3. Fisher BD, Strogatz DS. Community measures of low-fat milk consumption: comparing store shelves with households. Am J Public Health 1999;89(2):235-7.
4. Horowitz CR, Colson KA, Hebert PL, Lancaster K. Barriers to buying healthy foods for people with diabetes: evidence of environmental disparities. Am J Public Health 2004;94(9):1549-54.
5. Humpel N, Owen N, Leslie E. Environmental factors associated with adult's participation in physical activity: a review. Am J Prev Med 2002 Apr;22(3):188-99.
6. Morland K, Wing S, Diez-Roux A, Poole C. Neighborhood characteristics associated with the location of food stores and food service places. Am J Prev Med 2002;22(1):23-9.
7. Morland K, Wing S, Diez-Roux A. The contextual effect of the local food environment on residents' diets: the atherosclerosis risk in communities study. Am J Public Health 2002;92(11):1761-7.
8. Sallis J, Owen N. Physical activity and behavioral medicine. Thousand Oaks (CA): Sage Publications, 1999.
9. Sloane DC, Diamant AL, Lewis LB, Yancey AK, Flynn G, Nascimento LM, et al. REACH Coalition of the African American Building a Legacy of Health Project. Improving the food intake resource environment for healthy living through community-based participatory research. J Gen Intern Med 2003;18(7):568-75.
10. Taylor WC, Poston WSC, Jones L, Kraft MK. Environmental Justice. Obesity, physical activity, and healthy eating. J Phys Act Health 2006;3, S1, S30-39.
11. Trost SG, Owen N, Bauman AE, Sallis JF, Brown W. Correlates of adults' participation in physical activity: review and update. Med Sci Sports Exerc 2002;34(12):1996-2001.
12. Wechsler H, Basch CE, Zybert P, Lantigua R, Shea S. The availability of low-fat milk in an inner-city Latino community: implications for food intake education. Am J Public Health 1995;85(12):1690-2.

### Obesity Prevention and Treatment in African Americans

Shiriki Kumanyika, PhD, MPH
Professor of Epidemiology, Department of Biostatistics and Epidemiology
Associate Dean for Health Promotion and Disease Prevention
University of Pennsylvania School of Medicine
Philadelphia, Pennsylvania

Dr Kumanyika reviewed the prevalence of obesity in African American adults and children, pointing out that a majority of the adult population and a substantial proportion of children and adolescents are either overweight or obese. Yet, there are relatively few well-conducted studies of weight loss in African Americans and there is no consensus about the best way to address obesity in African American communities. Various types of cultural adaptations have been attempted. She described approaches taken in relatively large randomized trials of obesity prevention or treatment in which African Americans were included, noting that these approaches may actually yield larger weight losses in African Americans than those observed in culturally adapted studies. There are many differences between the large efficacy trials and the culturally adapted studies that can account for this, and it is inappropriate to compare these types of studies directly. Nevertheless, the impression from a review of the literature is that cultural adaptation, as currently defined, is not sufficient for achieving the needed level of weight control in African American communities. More important, the possibility exists that no individually focused intervention will be effective while the food availability, physical activity access, and psychosocial or cultural context variables, discussed throughout the workshop, are biased toward excess caloric intake and inadequate caloric expenditure. Interventions should be at multiple levels and include environmental and policy approaches.

Dr Kumanyika noted the particular lack of obesity prevention and treatment studies in African American children. Working with children around weight issues is in many ways more complex than working with adults. Assuring proper growth and avoiding stigmatization are key concerns. A weight-centered approach that is potentially harmful to children's self-esteem and their attitudes about eating is discouraged in favor of a health-centered approach focusing on the whole child, physically, mentally, and socially. Further, to the extent that children are less autonomous than adults in their behavioral choices, the environmental component of interventions in children and adolescents takes on more obvious significance. This includes home, school, and community environments. Dance has been a popular component of programs for African American children. Strategies to reduce television watching may also be effective. Television watching, which is higher in African American than white children and associated with excess calorie consumption as well as physical inactivity, is a prime target for weight control in African American children.

Dr Kumanyika concluded that obesity in African American communities is pervasive and well documented in national data. In contrast, the amount of informative research on effective intervention approaches is extremely small. New types of intervention approaches should be developed and evaluated.

**Related Readings**

1. Ard JD, Carter-Edwards L, Svetkey LP. A new model for developing and executing culturally appropriate behavior modification clinical trials for African Americans. Ethn Dis 2003;13(2):279-85.
2. Ard JD, Durant RW, Edwards LC, Svetkey LP. Perceptions of African-American culture and implications for clinical trial design. Ethn Dis 2005;15(2):292-9.
3. Baskin ML, Ahluwalia HK, Resnicow K. Obesity intervention among African-American children and adolescents. Pediatr Clin North Am 2001;48(4):1027-39.
4. Blocker DE, Freudenberg N. Developing comprehensive approaches to prevention and control of obesity among low-income, urban, African-American women. J Am Med Womens Assoc 2001;56(2):59-64.
5. Bronner Y, Boyington JE. Developing weight loss interventions for African-American women: elements of successful models. J Natl Med Assoc 2002;94(4):224-35.
6. Crawford PB, Story M, Wang MC, Ritchie LD, Sabry ZI. Ethnic issues in the epidemiology of childhood obesity. Pediatr Clin North Am 2001;48(4):855-78.
7. Kumanyika SK, Morssink C, Agurs T. Models for dietary and weight change in African-American women: identifying cultural components. Ethn Dis 1992;2(2):166-75.
8. Kumanyika SK. Obesity treatment in minorities. In: Wadden TA, Stunkard AJ, eds. Handbook of obesity treatment. New York: Guilford Publications, Inc.; 2002. p. 416-446.
9. Yancey AK, Kumanyika SK, Ponce NA, McCarthy WJ, Fielding JE, Leslie JP, et al. Population-based interventions engaging communities of color in healthy eating and active living: a review. Prev Chronic Dis 2004;1(1):A09. Epub 2003 Dec 15.

### Community Perspectives

Margaret Grayson
Board Chairperson
The Southeast Missouri Community Partners for Progress, Inc
Sikeston, Missouri

Anna Huff
Project Director
Mid-Delta Community Consortium of the Arkansas Rural Development Network
West Helena, Arkansas

The culminating activities of the workshop focused on clarifying research needs and perspectives. The tone was set by two presenters — Ms Margaret Grayson and Ms Anna Huff — who have been involved in community-based participatory research (CBPR). CBPR is "a collaborative research approach . . . designed to ensure and establish structures for participation by communities affected by the issue being studied, representative organizations, and researchers in all aspects of the research process, to improve health and well-being through taking action, including social change" (from Visnawathan et al, 2004). Inherent in CBPR are principles of co-learning and reciprocal transfer of expertise by all partners in the research, power-sharing in matters of decision-making, and mutual ownership of research processes and products. A recent review of 60 CBPR studies concluded that the level of community involvement "varied greatly," with 19 of 60 (32%) reporting shared funding, while community involvement mainly took the form of advisory committees, though there were some examples of communities as equal partners. One important finding, underscored by the presentations in this session, was that community involvement could enhance the research quality by improving relevance, participant recruitment, research methodology and dissemination, descriptive measures, and study outcomes.

These presentations demonstrated the importance of having direct accounts of CBPR by community partners as well as the more typical authorship by the academic partners. The importance of trust and respect among investigators and selected community members are key components for successful implementation of any community-based research. The ideal is for academic researchers to recognize community partners as equal partners, providing for respect and mutual benefit for community as well as university-based researchers, and for discussion of how the community at large will benefit from the project. Community-based interventions should be broad-based and include multiple components addressing the needs of different segments of the community and different stages in the life course. *Action research* is important both as a product of CBPR and a valued benefit to communities. Communities are "tired of being researched and studied and nothing happens" to improve the lives of community members.

The presenters stressed the importance of several aspects of adherence to CBPR principles. Power-sharing, shared decision-making, and community ownership are highly valued features of CBPR and should not be compromised if the intent is to foster long-term relationships between research partners. Respect for the knowledge of communities should be recognizable, as demonstrated through opportunities for partners from outside the community to hear the voices of community members, through a culture of co-learning where all partners can both learn and teach each other, and through direct research funding to community partners. Communities should be directly engaged from the inception of the project, with opportunities for continued involvement throughout to assure sustainability and ownership. However, this does not necessarily mean that communities want to "start from scratch." Community partners may be willing to try something that has already "proven" effective, since they want programs that work. Other essential considerations include logistical issues such as having flexible meeting schedules — times and accommodations (locations) that broaden opportunities for community member participation, community involvement in decisions about staffing and resource allocation, participation in/representation on governing bodies and in writing groups preparing publications. Capacity building should involve training in the research process for community members, covering subjects such as institutional review boards and human subjects' protection, certification and credentialing of data collectors, and opportunities for development of other skills and knowledge. Also, members of a community may recognize the need for multiple levels of impact at local, state, and federal levels and seek training in how to interact with political leaders.

**Related Readings**

1. Banks-Wallace, J, Enyart J, Lewis L, Lewis S, Parks L, Mitchell S, et al. Development of scholars interested in community-based health promotion research. West J Nurs Res 2002;24(4):326-44.
2. Fisher EB, Auslander W. Community organization and health promotion in minority neighborhoods. Ethn Dis 1992;2(3):252-72.
3. Green L, Mercer S. Can public health researchers and agencies reconcile the push from funding bodies and the pull from communities? Am J Pub. Health 2001;91:1926-9.
4. Institute of Medicine. Committee for the Study of the Future of Public Health. Div. Health Care Services. Future of public health. Washington (DC): National Academy Press;1988.
5. Israel B, Schulz A, Parker E, Becker, A. Review of community-based research: assessing partnership approaches to improve public health. Annu Rev Public Health 1998;19:173-202.
6. Minkler M. Ethical challenges for the "outside" researcher in community-based participatory research. Health Educ Behav 2004;31(6):684-97.
7. Smith MH. Community-based epidemiology: community involvement in defining social risk. J Health Soc Policy 1998;9(4):51-65.
8. Viswanathan M, Ammerman A, Eng E, et al. Community-based participatory research: assessing the evidence. Summary, Evidence Report/Technology Assessment: Number 99. AHRQ Publication Number 04-E022-1, August 2004. Rockville (MD): Agency for Healthcare Research and Quality. http://www.ahrq.gov/clinic/epcsums/cbprsum.htm
9. Williams RL, Yanoshik K. Can you do a community assessment without talking to the community? J Community Health 2001;26(4): 233-47.
